# Prevalence of mental disorders in young refugees and asylum seekers in European Countries: a systematic review

**DOI:** 10.1007/s00787-018-1215-z

**Published:** 2018-08-27

**Authors:** Christina Kien, Isolde Sommer, Anna Faustmann, Lacey Gibson, Martha Schneider, Eva Krczal, Robert Jank, Irma Klerings, Monika Szelag, Bernd Kerschner, Petter Brattström, Gerald Gartlehner

**Affiliations:** 1 Department for Evidence-based Medicine and Clinical Epidemiology, Danube-University Krems, Dr.-Karl-Dorrek Strasse 30, 3500 Krems an der Donau, Austria; 2 Department for Migration and Globalization, Danube-University Krems, Dr.-Karl-Dorrek Strasse 30, 3500 Krems an der Donau, Austria; 3 Department for Knowledge and Communications Management, Danube-University Krems, Dr.-Karl-Dorrek Strasse 30, 3500 Krems an der Donau, Austria; 4 Institute of Psychology, University of Graz, Universitätsplatz 3, 8010 Graz, Austria; 5 Department for Economy and Health, Danube-University Krems, Dr.-Karl-Dorrek Strasse 30, 3500 Krems an der Donau, Austria; 6 Department for Psychotherapy and Biopsychosocial Health, Danube University Krems, Dr.-Karl-Dorrek Strasse 30, 3500 Krems an der Donau, Austria; 7 RTI International, 3040 East Cornwallis Rd, Research Triangle Park, Durham, NC 27709 USA

**Keywords:** Accompanied refugee minors, Unaccompanied refugee minors, Posttraumatic stress disorder, Depression, Systematic review, Mental health

## Abstract

**Abstract:**

The European Union member states received about 385,000 asylum applications from children and adolescents below 18 years in 2015, and 398,000 in 2016. The latest political crises and war have led to an upsurge in refugee movements into European countries, giving rise to a re-evaluation of the epidemiology of psychiatric disorders and mental health problems among young refugees and asylum seekers. We systematically searched five electronic databases and reference lists of pertinent review articles. We then screened the results of forward citation tracking of key articles for relevant studies in the field for the period from January 1990 to October 2017. We dually reviewed citations and assessed risk of bias. We reported the results narratively, as meta-analyses were impeded due to high heterogeneity. We included 47 studies covered in 53 articles. Overall, the point prevalence of the investigated psychiatric disorders and mental health problems varied widely among studies (presenting interquartile ranges): for posttraumatic stress disorder between 19.0 and 52.7%, for depression between 10.3 and 32.8%, for anxiety disorders between 8.7 and 31.6%, and for emotional and behavioural problems between 19.8 and 35.0%. The highly heterogeneous evidence base could be improved by international, methodologically comparable studies with sufficiently large sample sizes drawn randomly among specific refugee populations. The prevalence estimates suggest, nevertheless, that specialized mental health care services for the most vulnerable refugee and asylum-seeking populations are needed.

**Registration:**

The systematic review protocol was registered in PROSPERO on October 19th, 2017 with the number: CRD42017080039 and is available from: https://www.crd.york.ac.uk/prospero/display_record.php?RecordID=80039

**Electronic supplementary material:**

The online version of this article (10.1007/s00787-018-1215-z) contains supplementary material, which is available to authorized users.

## Introduction

European countries faced unusually high arrivals of asylum seekers mainly from Syria, Afghanistan, and Iraq in 2015 and 2016, with Austria, France, Germany, Greece, Italy, Sweden, and United Kingdom as main destination countries [1]. With 1.3 million arrivals in both 2015 and 2016, the number of asylum applications in the European Union (EU) far exceeded the previous peak of about 672,000 in 1992, when the EU member states received many asylum seekers from former Yugoslavia [2].

Within this group, the most vulnerable asylum seekers and refugees are children and adolescents. In legal terms, asylum seekers are individuals who have applied for asylum on an individual basis and whose claim is pending. Refugees are individuals recognized based on the United Nations Refugee Convention, which gives them a legal residence status [3]. During the last years, increasing numbers of unaccompanied refugee minors (URM) (i.e. children under the age of 18, who came to the host country without a parent or other caregiver) as well as children and adolescents accompanied by their parents or caregivers, were seeking asylum in different countries worldwide [4], as well as in the EU. Overall, unaccompanied or accompanied minors represent one-third of all asylum seekers in the EU [1]. In the year 2015, around 385,000 children and adolescents below 18 years sought asylum in the EU. In the year 2016, this number rose to 398,000, and in 2017, the number rose again to 213,000 [5].

Forced migration sets children and adolescents under increased challenges that compound the usual developmental challenges of childhood. For example, before or during the forced migration, minors might have experienced war or death of relatives and friends and might have been exposed to violence. Furthermore, these children and adolescents might be confronted with different challenges such as adaptation processes to the new setting, racial discrimination, and complex legal immigration processes [6]. These stressors experienced in the country of origin, during migration as well as in the host country, may lead to strong psychological pressure and physical challenges and therefore affect children’s and adolescents’ physical health and well-being [7]. Studies show that traumatic and ongoing adverse experiences as well as social risk factors can lead to mental stress disorders, in specific posttraumatic stress disorders (PTSD), depression, anxiety disorders or psychological distress (e.g. unspecified mental disorders, externalizing behaviour) [8–11]. In addition, long-lasting asylum procedures with uncertain outcomes represent high psychological burdens [6]. Even after a long period in the host country, mental stress remains high [12].

These findings highlight the special vulnerability of both accompanied and unaccompanied refugee children and adolescents in terms of mental health. To take planning steps for guaranteeing an effective mental health care, it is important to be aware of the mental health status of refugee populations in general [13, 14]. The two most recent systematic reviews on the prevalence of psychiatric disorders in asylum-seeking or refugee children and adolescents included studies up to 2008 [15] and 2015, respectively [16]. Since then, new or re-emerging political crises and war have led to an upsurge in refugee movements into European countries, giving rise to a re-evaluation of the prevalence of mental disorders among young refugees. Furthermore, both systematic reviews have methodological limitations that could have had an impact on the completeness of the included evidence base and the results.

Therefore, the goal of our project was to conduct an up-to-date systematic review guided by the Joanna Briggs Institute Reviewers’ Manual for protocols and systematic reviews as this is—to our best knowledge—the only available guideline for systematic reviews on prevalence data [17], addressing the following research question: What is the prevalence of mental health disorders among asylum-seeking or refugee children and adolescents (either accompanied or unaccompanied) in European countries?

## Methods

We developed a systematic review protocol according to the Preferred Reporting Items for Systematic Reviews and Meta-Analysis for Protocols (PRISMA-P) reporting guidelines [18]. The research question laid out in this article was part of a systematic review registered in PROSPERO (an international database of prospectively registered systematic reviews in health and social care) on October 19th, 2017 under the number: CRD42017080039. Throughout this manuscript, we followed the PRISMA (Preferred Reporting Items for Systematic Reviews and Meta-Analyses) statement [19] to report this systematic review.

### Eligibility criteria

In accordance with the research question, we specified a priori a list of inclusion and exclusion criteria following the recommended Condition-Context-Population-Framework (CoCoPop) for prevalence data [17]. We included studies if they (1) were published in peer-reviewed journal articles, (2) investigated the prevalence of psychiatric disorders (as primary outcomes) or other psychological problems (as secondary outcomes), (3) examined unaccompanied or accompanied asylum-seeking children and adolescents or refugee minors (≤ 21 years, to allow for some outliers in the age group), (4) and were conducted in European countries. We excluded studies, which provided data on internally displaced children only or on children and adolescents that were invited to participate in the study after their attendance of a mental health clinic. The detailed inclusion and exclusion criteria are depicted in Table 1. We are not reporting on differences between refugees and asylum seekers because most studies included mixed populations or did not state explicitly the legal status of the included children and adolescents.

**Table 1:** Eligibility criteria according to Condition-Context-Population-Framework (CoCoPop) for prevalence data [17]

	Inclusion criteria	Exclusion criteria
Condition	Primary outcomes: anxiety disorder (general anxiety disorder, social phobia, panic disorder), bipolar disorder, eating disorder, major depressive disorder, posttraumatic stress disorder, schizophrenia, somatoform disorders, substance abuse of illicit drugs, alcohol and tobacco, suicidal ideation and behaviour Secondary outcomes: *Other psychological problems*: psychological distress, behaviour problems, mental health problems, emotional problems, emotional well-being	All other mental disorders, such as personality disorder
	Assessment process: With a reliable, validated self-assessment or proxy-assessment tool or with a structured clinical interview Assessment time: When the assessment happened over several time points we took the first available data point	Self-developed questionnaire without reporting on psychometric properties
Context	European Countries (Albania, Andorra, Armenia, Austria, Azerbaijan, Belarus, Belgium, Bosnia and Herzegovina, Bulgaria, Croatia, Cyprus, Czech Republic, Denmark, Estonia, Finland, France, Georgia, Germany, Greece, Hungary, Iceland, Ireland, Italy, Kazakhstan, Kosovo, Latvia, Liechtenstein, Lithuania, Luxembourg, Macedonia [FYROM], Malta, Moldova, Monaco, Montenegro, Netherlands, Norway, Poland, Portugal, Romania, Russia, San Marino, Serbia, Slovakia, Slovenia, Spain, Sweden, Switzerland, Turkey, Ukraine, United Kingdom [UK], Vatican City [Holy See]) see list: https://www.countries-ofthe-world.com/countries-of-europe.html	
Population	Unaccompanied or accompanied asylum-seeking children and adolescents or refugee minors (≤ 21 years) [including adolescents up to 23 years, if the age group 21–23 years comprised < 50% of the study population]	Internally displaced children and adolescents (i.e. people who have not crossed a boarder to find safety); Study participation is based on the attendance of a mental health department/clinic

### Literature search strategy

We conducted a systematic literature search of Ovid MEDLINE, PsycINFO (via Ebsco), CINAHL (The Cumulative Index to Nursing and Allied Health Literature, via Ebsco), PubMed (for non-MEDLINE content) and Scopus for the period from January, 1st 1990 to October, 17th 2017. An experienced information specialist developed a search strategy using a combination of different MeSH (Medical subject headings) terms and variations of free-text key words (consisting of search terms for ‘refugees’ AND ‘minors’ AND ‘mental disorders’ AND ‘Europe’). We did not limit the search to any specific languages. The search strategy was developed in Ovid MEDLINE and adapted for the other databases. The detailed search strategy, which we tested using known relevant articles, is available in Additional Material 1. Additionally, we complemented electronic searches by checking reference lists of pertinent review articles and using forward citation tracking of key articles in the field. We further conducted grey literature searches considering government surveillance data, reports from World Health Organization (WHO), United Nations High Commissioner for Refugees (UNHCR), the European Council on Refugees and Exiles (ECRE) and Médecins Sans Frontières (MSF).

### Study selection and data extraction

We imported all references into the Systematic Review Software Covidence [20] and used this program throughout the review process. Two reviewers independently screened abstracts and full-texts against pre-specified criteria. They resolved discrepancies about inclusion or exclusion by consensus or by involving a third reviewer. The abstract and full-text review forms were pilot-tested and the review form was adapted according to feedback of the pilot review participants.

Next, we pilot-tested and used standardized data extraction forms to gather relevant information systematically from each study. One investigator extracted data relating to study information, study method, population, and outcomes/condition. A second investigator checked all extracted data for completeness and accuracy. If the publications did not report the necessary information for relevant outcomes, we contacted authors to request additional data.

### Risk of bias assessment of selected studies

We evaluated the risk of bias of included studies using a modified version of the standardized critical appraisal tool (AXIS tool) to assess the quality and risk of bias in cross-sectional studies [21]. Risk of bias was assessed at the outcome level by two independent reviewers. Again, the reviewers solved disagreement by discussion or by involving a third reviewer. We collapsed the information into a final risk of bias rating for each individual included study using three distinct categories: low, unclear, or high risk of bias.

### Synthesis and analysis

We performed exploratory random effect meta-analyses based on a logit transformation to calculate weighted summary proportions using generic inverse variance models to synthesise prevalence estimates of studies that were similar with respect to outcome measurement [17]. We pooled studies when there were at least three different studies reporting on the same outcome. We tested for heterogeneity with Cochrane’s Q test and quantified its magnitude using *I*^2^ and *τ*^2^. We conducted all statistical analyses using Comprehensive Meta-Analysis version 3.0.

Because of high heterogeneity for all outcomes of interest, we conducted subgroup analyses to explore heterogeneity: (i) accompanied vs. unaccompanied refugees/asylum seekers, (ii) type of assessment of mental disorders (structured clinical interview vs. self-report questionnaires), and (iii) sampling procedure (random sampling or census survey vs. non-random sampling). As we did not identify enough similar studies—contrary to what we stated in the protocol—we could not analyse differences between host countries and between placement types (refugee camps vs. non-refugee camps). We carried out sensitivity analyses to assess the effect of high risk of bias studies on the overall effect. Because of very high, unexplained heterogeneity for all outcomes of interest, we do not report pooled effects of prevalence estimates. Throughout the manuscript, we present prevalence estimates as medians and interquartile ranges. Interquartile ranges reflect estimates between the 25th and the 75th percentiles of a range of estimates and eliminate extreme values at both ends of a spectrum.

### Confidence in cumulative evidence

For assessing the confidence in available evidence for the prevalence of psychiatric disorders, we followed the guidance of the Project on a Framework for Rating Evidence in Public Health (PRECEPT) [22]. This project adapted the GRADE scheme (Grading of Recommendations Assessment, Development and Evaluation) [23] to prevalence data [22]. We only considered the defined primary outcomes but not the secondary outcomes stated in the protocol (see Table 1). No decision-makers were involved in the selection of outcomes. Criteria for downgrading the quality of evidence according to GRADE are risk of bias, inconsistency, indirectness, imprecision and publication bias using four grades: high, moderate, low, and very low. For each outcome, the quality of evidence is initially rated as “high” and not as “low” as in other observational studies [22]. We dually evaluated both the overall quality of evidence for each outcome and solved disagreement through discussion.

## Results

### Results of the search strategy

**Fig. 1:**
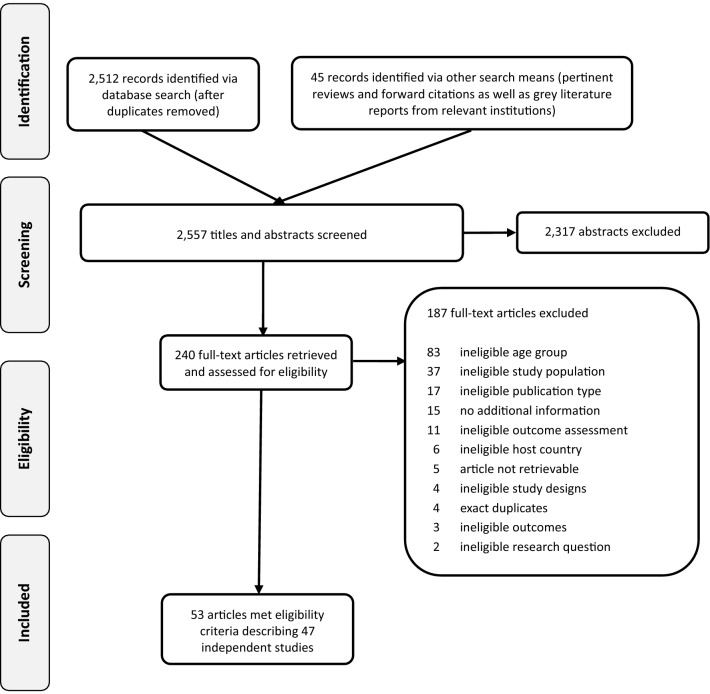
PRISMA flow diagram of the study selection process

Overall, we identified 2557 citations from searches and reviews of reference lists after removal of duplicates. Of these, we assessed 240 full-texts for eligibility. Finally, we included 47 studies reported in 53 articles [24–76] (see Fig. 1). 15 additional articles were accompanying articles, but did not reveal any further information relevant for our research question and were therefore excluded [8, 9, 11, 77–88]. We obtained additional data for eleven studies after contacting authors [28, 48, 53, 60, 62, 64, 66, 67, 73–75]. Seven studies were published in other languages than English: Danish [55, 69], German [48, 59, 75, 76] and Italian [65]. We translated the relevant information into English. Overall, ten studies [26, 31, 40, 45, 47, 51, 58, 68, 70, 73] reported mean and standard deviation for relevant outcomes. These results are presented in Additional Material 2.

### Description of included studies

Table 2 provides an overview of the included studies summarizing population characteristics, data collection methods and outcomes. Studies reported information on 24,786 refugee or asylum-seeking children and adolescents living in 14 European countries (Austria, Belgium, Croatia, Denmark, Finland, Germany, Greece, Italy, Netherlands, Norway, Slovenia, Sweden, Turkey, and United Kingdom). One large register-based study already contributed data on 15,264 children and adolescents [26]. Overall, the studies included more boys than girls. The mean age of the participants varied between 5.9 and 18.6 years (range 2–23 years). Seven studies assessed the children’s and adolescent’s mental health after 2010, 13 studies were conducted between 2000 and 2010 and eight studies were conducted earlier. The length of stay in the host country ranged from 4 months to 9 years. The countries of origin varied widely with the majority of study participants coming from Africa, Afghanistan, Iran, Iraq, and countries from former Yugoslavia. Two studies [26, 57] received a low risk of bias rating and 29 studies [24, 25, 27, 30, 33–39, 41, 42, 44–51, 54, 56, 61, 62, 66, 68, 75, 76] received an unclear risk of bias rating (see Table 2). Reasons for unclear risk of bias ratings were mainly because the sample was a convenience sample, the sample size was not justified and rather small, or the survey’s response rate was not reported. Sixteen studies showed high risk of bias [29, 32, 52, 55, 58–60, 63, 65, 67, 69–74], because of low response rates (< 30%), inconsistent results, or the lack of informed consent or ethical approval (as rated by the AXIS tool).

**Table 2:** Study characteristics of included studies

Study	Country of study	Region of origin: *n* (%)^†^	Sample size, *n*	Sampling procedure	Year of assessment	Mean age in years, M (SD)	Gender—female, *n* (%)	*u*/*a*	Outcome	RoB
Adam and Klasen [67]	Germany	ME: NR (80.9%) EE: NR (19.1%)	215	NRS	2002–2003	14.8 (2.1)	NR (41.4%)	*a*	A, D, EBP, PTSD	High
Almqvist et al. [24]	Sweden	ME: 50 (100%)	50	NRS	NR	M: 5.8 (1.1)	14 (28%)	*a*	PTSD	Uncl
						F: 5.8 (1.1)				
Angel et al. [25]	Sweden	EE: 99 (100%)	99	NRS	1994–1995	11.3 (NR)	NR	*a*	A, EBP, PTSD	Uncl
Barghadouch et al. [26]	Denmark	AS: 2349 (15.4%) EE: 6505 (42.6%)	15,264	Census	NR	9.1 (NR)	6909 (45.3%)	*u* and *a*	AD^*^, ND^*^, PD^*^	Low
Bean et al. [27, 28]	Netherlands	AF: NR (53%) AS: NR (8%) ME: NR (4%)	1110	NRS/RS	2002	15.8 (NR)	329 (29.8%)	*u*	A, D, EBP, PTSD	Uncl
Begovac et al. [29]	Germany/Croatia	EE: 133 (100%)	133	NRS	1997–1999	NR	160 (49.7%)	NR	D, PTSD	High
Bronstein et al. [30]	UK	ME: 222 (100%)	222	NRS	NR	16.3 (1.0)	0 (0%)	*u*	A, D, PTSD	Uncl
Derluyn et al. [32]	Belgium	AF: NR (59%) AS: NR (25.3%) EE: NR (14.3%) SA: 2 (1.2%)	142	NRS	NR	<14 y: 9.6% 15–17 y: 52.9% >18 years 37.3%	NR (37.3%)	*u*	A, D, EBP	High
Derluyn et al. [33]	Belgium	AF: 517 (43.4%) AS: 267 (22.4%) EE: 349 (29.3%) SA: 59 (4.9%)	1234	RS	2002–2003	16.6 (1.31)	U: 43 (35.8%) A: 516 (46.5%)	*u* and *a*	A, D, PTSD	Uncl
Elklit et al. [34]	Denmark	EE: 119 (100%)	119	NRS	NR	18.5 (1.8)	NR (33.3%)	*u* and *a*	PTSD	Uncl
Fazel et al. [35]	UK	EE: NR (48%) AS: NR (16%) ME: NR (10%)	101	NRS	NR	NR	40 (40%)	*u* and *a*	EBP	Uncl
Gavranidou et al. [68]	Germany	EE: 32 (NR) ME: 11 (NR)	55	NRS	NR	13.4 (NR)	23 (41.8%)	*u* and *a*	A^*^, EBP^*^	Uncl
Goldin et al. [36]	Sweden	EE: NR	48	NRS	1994–1995	NR	24 (50%)	*a*	A, D, PTSD, HYP, PSYCom	Uncl
Gusic et al. [72]	Sweden	ME: 26 (60%)	42	NRS	NR	16.1 (1.5)	16 (38%)	*u* and *a*	DP, PTSD	High
Hjern et al. [37]	Sweden	SA: 43 (68%) ME 20 (32%)	63	NRS	1986–1987	5.9 (NR)	NR	*a*	EBP	Uncl
Hodes et al. [38]	UK	~EE: 34 (28.8%) AF: 52 (44.1%) ME: 26 (22.0%) AS: 3 (2.5%) SA: 3 (2.5%)	113	NRS	2002–2003	17.0 (NR)	44 (38.9%)	*u* and *a*	D, PTSD	Uncl
Hollins et al. [71]	UK	EE: 99 (100%)	99	NRS	NR	16 (1.2)	12 (NR)	*u*	A, EBP	High
Huemer et al. [39]	Austria	AF: 41 (100%)	41	NRS	NR	17.0 (0.8)	6 (15%)	*u*	A^*^, D, PTSD, S	Uncl
Jakobsen et al. [61]	Norway	ME: 105 (76%) AF: 33 (24%)	138	NRS	2009–2011	16.2 (0.8)	0 (0%)	*u*	A, D, EBP, OPD, PTSD	Uncl
Jensen et al. [41]	Norway	~AS: 59 (63%) AF: 34 (37%) EE: 1 (1%)	93	Census	2010–2012	13.8 (1.4)	28 (19%)	*u*	A, D, EBP, PTSD	Uncl
Kocijan-Hercigonja et al. [70]	Croatia	EE: 35 (100%)	35	NRS	NR	NR	NR	*a*	A^*^, D^*^, PSYCom^*^	High
Longobardi et al. [74]	Italy	AF: 12 (63.2%) EE: 5 (26.3%) AS: 1 (5.3%)	23	NRS	NR	NR	1 (5%)	*u*	A, D, PTSD	High
Möhrle et al. [75]	Germany	ME: NR (51.6%) AF: NR (16.8%)	191	NRS	2015–201	17.1 (1.2)	0 (0%)	*u*	EBP	Uncl
Montgomery [42]	Denmark	ME: 131 (96.24%)	131	NRS	2000–2001	15.3 (NR)	76 (58%)	*u* and *a*	EBP	Uncl
Nasiroglu and Ceri [60]	Turkey	ME: 55 (100%)	55	NRS	NR	11 (3.7)	25 (45.6%)	NR	A, D, HYP, NE, PTSD	High
Nielsen et al. [44]	Denmark	EE: 118 (48%) ME: 67 (27%)	246	NRS	2006	9.6 (NR)	104 (42%)	*a*	EBP	Uncl
Oppedal and Idsoe [45]	Norway	ME: NR (70%)	948	NRS	2000–2010	18.6 (2.5)	NR (15.8%)	*u*	D^*^, PTSD	Uncl
Papageorgiou et al. [46]	Greece	EE: NR	95	NRS	NR	9.6 (NR)	54 (57%)	*u*	A, D, EBP, OPD, PTSD	Uncl
Reijneveld et al. [47]	Netherlands	EE: 6 (4.9%) AS: 9 (7.4%) AF: 107 (87.7%)	122	NRS	2002 –2003	16.1 (0.7)	40 (32.8%)	*u*	A^*^, D^*^, EBP^*^, PTSD^*^	Uncl
Reis et al. [48]	Germany	ME: 40 (80%)	58	NRS	2007–2009	12 (NR)	25 (43%)	*a*	EBP	Uncl
Rücker et al. [59]	Germany	ME: 45 (86.5%) OC: 7 (13.5%)	52	NRS	2016	16.2 (1.4)	1 (1.9%)	*u*	EBP	High
Ruf et al. [76]	Germany	EE: 47 (45.2%)	98	NRS	2003–2004	10.6 (2.6)	56 (53.8%)	*a*	D, S, PTSD	Uncl
Sabuncuoglu and Berkem [73]	Turkey	EE: 19 (100%)	19	NRS	NR	Range 13–73 (NR)	10 (53%)	*u* and *a*	D^*^	High
Salari et al. [49]	Sweden	ME: NR (100%)	208	NRS	2015–2016	15.4 (1.3)	NR (2.4%)	*u*	PTSD	Uncl
Sanchez-Cao et al. [50]	UK	EE: 25 (35%) AF: 36 (51%)	71	NRS	2003–2004	NR	23 (32.4%)	*u*	D, EBP, PTSD	Uncl
Sikic et al. [51]	Croatia	EE: NR	669	NRS	1994–1995	12.03 (2.5)	989 (48%)	NR	A^*^, D^*^	Uncl
Sleijpen et al. [62]	Netherlands	ME: 52 (46.8%) AS: 15 (13.5%) AF: 6 (5.4%) OC: 37 (33.3%)	111	RS	2014–2015	14.5 (1.8)	57 (51%)	NR	PTSD	Uncl
Slodnjak et al. [52]	Slovenia	EE: NR	265	NRS	1994	NR	140 (52.8%)	*u* and *a*	D, S, PTSD	High
Sourander et al. [54]	Finland	AF: 42 (91.3%)	46	NRS	NR	14.1 (2.3)	12 (NR)	*u*	EBP	Uncl
Staehr [69]	Denmark	EE: NR	232	NRS	1999	NR	NR	NR	PTSD	High
Staehr et al. [55]	Denmark	NR	780	NRS	2001 –2003	NR	NR	*u* and *a*	S	High
Stotz et al. [63]	Germany	NR	32	NRS	2011	15.6 (2.2)	0 (0%)	*u* and *a*	PTSD	High
Taurino et al. [65]	Italy	AF: 29 (87%)	34	NRS	NR	18.1 (0.6)	0 (0%)	*u*	A, D, PTSD	High
Thommessen et al. [66]	Italy	AF: 16 (NR) ME: 15 (NR) AS: 29 (NR)	60	NRS	NR	NR	0 (0%)	*u*	EBP	Uncl
Vervliet et al. [56]	Belgium/Norway	ME: 212 (69.1%) AF: 92 (30.0%) AS: 2 (0.6%) EE: 1 (0.3%)	307	Census	NR	16.1 (0.8)	16 (5.2%)	*u*	A, D, PTSD	Uncl
Wiegersma et al. [57]	Netherlands	ME: NR (37%) EE: NR (39%) AF: NR (24%)	267	NRS	2003	9.9 (3.5)	NR (50%)	*a*	EBP	Low
Yurtbay et al. [58]	Turkey	EE: 250 (100%)	250	NRS	1999	Children: 10.8 (1.47) Adolescents: 15.4 (1.43)	125 (50%)	NR	A, D^*^	High

*A* anxiety, *a* accompanied, *AD* affective disorder, *AF* Africa, *AS* Asia, *D* depression, *DP* dissociative psychopathology, *EBP* emotional and behavioural problems, *EE* Eastern Europe, *HYP* hyperactiveness, *M* mean, *ME* Middle East, *n* sample size, *NE* nocturnal enuresis, *ND* neurotic disorder, *NR* not reported, *NRS* non-random sampling, *OPD* overall psychiatric disorder, *PD* psychotic disorder, *PSYCom* psychiatric complaints, *PTSD* posttraumatic stress disorder, *RoB* risk of bias, *RS* random sampling, *S* suicidal ideation and behaviour, *SA* South America, *SD* standard deviation, *u* unaccompanied, *Uncl* unclear

^*^mean and standard deviation but no point prevalences are reported

^~^overall sample size differed from *n* of distribution of countries of origin

^†^we grouped the reported countries of origin into Asia, Africa, Eastern Europe, Middle East and South America

Unexplained high heterogeneity hampered the conduct of meta-analyses for all outcomes (PTSD: *I*^2^ = 96%; depression: *I*^2^ = 94%; anxiety disorder: *I*^2^ = 96%; suicidal ideation and behaviour: *I*^2^ = 90%; emotional and behavioural problems: *I*^2^ = 87%;). See Additional Material 3 for point prevalence and confidence interval (CI) for outcomes of individual studies.

**Fig. 2:**
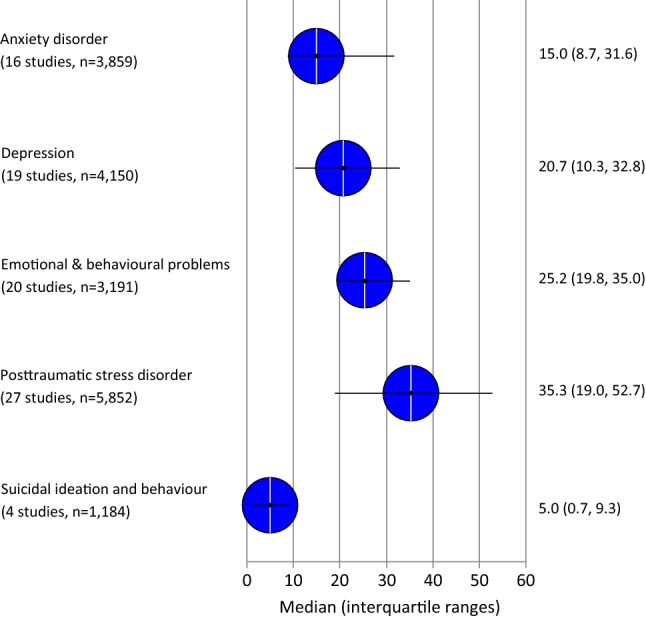
Median and interquartile ranges of point prevalences of psychiatric disorders and mental problems in young refugees and asylum seekers. *n* sample size

Figure 2 presents an overview of the median point prevalence and the interquartile ranges of the relevant outcomes. These results are described in more detail below.

### Posttraumatic stress disorder

Twenty-seven studies reported on the prevalence of PTSD, providing data on a total of 5852 young refugees or asylum seekers [24, 25, 27, 29, 30, 33, 34, 36, 38, 39, 41, 45, 46, 49, 50, 52, 56, 60–63, 65, 67, 69, 72, 74, 76]. The interquartile range (IQR) for refugees and asylum seekers screening positive for PTSD was 19.0–52.7% and the median was 35.3%. The two largest studies rated with unclear risk of bias reported a PTSD prevalence of 42.3% (95% CI 39.1%, 45.6%, *n* = 875) [53] and 52.0% (95% CI 48.7%, 55.2%, *n* = 895) [45]. PTSD was screened via self-report questionnaires (Impact of War-related Trauma Events [IWRITE], Reactions of Adolescents to Traumatic Stress Questionnaire [RATS]).

Generally, studies relying on clinical interviews for the diagnosis of PTSD showed a lower PTSD prevalence than studies using self-report or proxy-questionnaires (IQR 19.2–36.0% vs. 25.6–62.7%). Three studies directly compared differences between unaccompanied and accompanied children and adolescents. They reported a higher prevalence for being screened positive for PTSD or to be diagnosed with PTSD among unaccompanied than accompanied children and adolescents (Study 1 [different accompanied groups]: 36.7% vs. 5.7–12.7% [33]; Study 2 [gender]: 61.5–73.1% vs 14.3–35.3% [38]; and Study 3: 50% vs. 0% [63]).

Overall, the quality of evidence regarding the prevalence of PTSD in refugee and asylum-seeking children and adolescents is low. This is because the evidence relies mainly on studies that used convenience sampling strategies (downgraded for risks of bias), and the studies’ results varied considerably (downgraded for inconsistency).

### Depression

Nineteen studies, including 4150 children and adolescents, analysed the prevalence of depression [27, 29, 31–33, 36, 38, 39, 41, 46, 50, 52, 56, 60, 61, 65, 67, 74, 76]. The IQR for depression prevalence was 10.3–32.8% and the median was 20.7%. As for PTSD, studies using a structured clinical interview (e.g. M.I.N.I Kid) as assessment method reported lower percentages of depression 3.1% to 9.4% [39, 61, 76] compared to the median of all assessments (20.7%).

The two largest studies both using the Hopkins Symptom Checklist-37A [HSCL-37A] (a self-report questionnaire for the screening for depression) revealed very different results. In a sample of 650 accompanied refugees and asylum seekers in Belgium, the study reported a depression prevalence of 8.2% (95% CI 6.3%, 10.5%) [33]. By contrast, a study on 827 unaccompanied children and adolescents in the Netherlands [28] reported a prevalence for depression of 38.8% (95% CI 35.6%, 42.2%). The Belgian study used a higher cut-off-point (90th percentile instead of the 60th percentile), which resulted in a lower prevalence. Two studies directly comparing differences between unaccompanied and accompanied children and adolescents tended to show higher point prevalence for depression for unaccompanied children and adolescents than for accompanied children (30.2% vs. 8.2–32.8% [different accompanied groups] [33] and 11.5–23.1% vs. 5.9–52.9% [gender] [38]).

Overall, the quality of evidence regarding the prevalence of depression in refugee and asylum-seeking children and adolescents is low (downgraded for risk of bias and inconsistency).

### Anxiety disorder

We identified 16 studies including 3804 children and adolescents that assessed the point prevalence of anxiety disorders [25, 27, 30, 32, 33, 36, 41, 46, 56, 58, 60, 61, 65, 67, 71, 74]. The IQR for the anxiety disorder prevalence was 8.7–31.6% and the median was 15.0%. The largest study investigating 852 unaccompanied minors in the Netherlands [28] reported a point prevalence of anxiety disorder of 46.0% (95% CI 42.7%, 49.4%). Bronstein et al. [31] applied the same questionnaire (HSCL-37A with the same cut-off point) as Bean et al. [28] in a similar war-affected unaccompanied population from Afghanistan living in the UK (*n* = 214), but reported a lower prevalence of anxiety disorder, 34.6% (95% CI 28.5%, 41.2%). The differences in these results could be due to differences in the host country, sampling errors or gender differences; while Bronstein et al. [31] included only boys, Bean et al. [28] included boys (70%) and girls (30%). One study directly comparing differences between unaccompanied and accompanied children and adolescents, showed higher anxiety disorder prevalence in unaccompanied children (20.2%; 95% CI 14.0%, 28.1%) than in accompanied children (8.8%; 95% CI 6.8%, 11.2%) [33].

Overall, the quality of evidence regarding the prevalence of anxiety disorder in refugee and asylum-seeking children and adolescents is low (downgraded for risk of bias and inconsistency).

### Suicidal ideation and behaviour

Four studies [39, 52, 55, 76] reported on suicidal ideation and behaviour of 1184 children and adolescents revealing a median of 5.0% and an IQR of 0.7–9.3%. Unclear risk of bias studies [39, 76] relying on the assessment of 139 children and adolescents using a clinical interview (M.I.N.I. Kid) revealed higher prevalence (9.2% and 9.8%) than the two high risk of bias studies. A Danish register-based study [55] assessed suicide attempts (0.9%, 95% CI 0.4%, 1.9%) and a Slovenian study investigated “the intention to kill oneself” (0.8%, 95% CI 0.2%, 3.0%) in a sample of refugee adolescents [52].

Overall, the quality of the evidence regarding the prevalence of suicidal ideation and behaviour of refugee and asylum-seeking children and adolescents is very low (due to risk of bias, imprecision and inconsistency).

### Emotional and behavioural problems

Overall, 20 studies covering 3191 refugee or asylum-seeking children and adolescents reported on emotional and behavioural problems assessed with eight different self-report or proxy-questionnaires [25, 27, 30, 32, 35, 37, 41, 42, 44, 46, 48, 50, 54, 57, 59, 61, 66, 67, 71, 75]. The IQR of the point prevalence of emotional and behavioural problems is 19.8–35.0% and the median is 25.2%. The largest study [28] reported on prevalence of emotional and behavioural problems in 41.2% (95% CI 37.9%, 44.6%) of the participating unaccompanied children and adolescents living in the Netherlands.

### Other outcomes

Few studies [36, 60] reported on point prevalence of other outcomes, such as hyper activeness (1.8–6%) or psychosomatic complaints (13%) [36], any psychiatric disorders as an overall category (13.4–41.9%) [46, 61], nocturnal enuresis (10.9%) [60], and dissociative psychopathology (36%) [72]. We could not identify any studies reporting on the prevalence of bipolar disorder, eating disorder, schizophrenia, and substance abuse of illicit drugs, alcohol and tobacco.

## Discussion

Overall, the point prevalence of the investigated psychiatric disorders and mental health problems varied widely and showed that up to a third of refugee and asylum-seeking minors could be affected by either a depression or anxiety disorder or by any other emotional or behavioural problem. Furthermore, up to half of refugee and asylum-seeking minors could be affected by PTSD (IQR: 19.0–52.7%). In general, in comparison to the most up-to-date systematic review on this topic [16], we were able to identify ten additional studies. Nevertheless, the results were within a comparable range [15, 16].

In comparison to the general population, the prevalence of psychiatric disorders and mental health problems is substantially higher. As reported in a systematic review including 48 studies conducted in 27 countries and published from 1985 to 2012 and including a sample size of approximately 90,000 children and adolescents, the estimated worldwide point prevalence of diverse psychiatric disorders for children and adolescents [89] were reported to be lower than the above-mentioned results: The prevalence of any depressive disorder was 2.6% (95% CI 1.7%, 3.9%) and any anxiety disorder was 6.5% (95% CI 4.7%, 9.1%). The prevalence of suicidal behaviour (as a lifetime prevalence) in an Austrian mental health study was 1.8% (95% CI 0.6%, 3.0%) [90]. Furthermore, direct comparisons in our included studies showed that in general, refugee and asylum-seeking children and adolescents were to a greater extent affected by depression, anxiety, and emotional and behavioural problems [27, 40, 51, 68, 70] than native children and adolescents. These differences may be influenced by the different traumatizing experiences in the home country or during migration and diverse challenges or problems in the host country [6]. The literature highlights several pre-migration (e.g. exposure to poverty, violence, war and war-like conditions, but also acquired education, family, social and cultural values), peri-migration (e.g. exposure to traumatic events such as separation, sexual abuse, and trafficking) and post-migration (e.g. access to schooling, social support, stable settlement, parents mental health, insecurity during legal aspects of the immigration process, cultural adaptation) factors influencing the point prevalence of psychiatric disorders in children and adolescents [6, 47, 91].

Participants from most studies came from countries with wars (e.g. African states such as Somalia, Afghanistan, Balkan states), which could explain why prevalence estimates of PTSD (median: 35.5%, range 19.0–52.7%) were higher than for the other disorders. Our analyses also showed that unaccompanied minors revealed a higher risk of suffering from PTSD, depression, and anxiety disorders [33, 38, 63] than accompanied minors, which highlights the need for a special protection of this group. The association and complex interplay of migration factors with mental health disorders does, however, require an in-depth understanding which cannot be provided by the results of this review. The review rather provides a snapshot on mental health prevalence of different periods (over 30 years) in the younger European history.

Our systematic review showed a high heterogeneity of individual studies. This high variability of results was also found in other systematic reviews [92] and may be the result of several influencing methodological and clinical factors. From a methodological perspective, the included studies varied according to sample sizes, sampling strategies (random sampling or census studies versus non-random sampling), and assessment procedures. Structured clinical interviews relying on standard diagnostic criteria (DSM in different versions) tended to result in lower point prevalence in comparison with self-report or proxy-report questionnaires [93] (see Additional Material 4 for a list of the included instruments). These questionnaires may rather be seen as screening instruments that give an indication of a potential problem requiring further psychological or psychiatric investigation [15]. Furthermore, self-report questionnaires were sometimes used with different cut-off-values (e.g. SDQ [35, 44, 75] or the RATS [30, 33, 53]. From a clinical perspective, the studies comprised individuals from diverse social and cultural backgrounds residing in different host countries with varying legal and official support systems and asylum polices. It has recently been shown that the Western-based approach to psychiatric assessment simplifies the complex issue of migration [94].

Assessment and diagnosis difficulties could also explain why our review identified only very limited evidence on psychosomatic complaints. Questionnaires assessing psychosomatic complaints have shown to have moderate agreement with interviews, which are in themselves hampered by difficulties of patients to locate the discomfort or pain [95] and expectations to be treated for somatic symptoms on both patient’s and physician’s side [96]. Various symptoms have reportedly been associated with mental health disorders including constipation, amenorrhea or a dry mouth with depression, diarrhoea or hyperhidrosis with anxiety disorders, and quick respiration, palpitations, hyperhidrosis, and pain with PTSD [96]. Future studies should address the under-researched issue of psychosomatic complaints among asylum-seeking and refugee children and adolescents [6, 33, 38, 47, 63].

Overall, results of our study highlight the special attention that should be given to the mental health problems of refugee and asylum-seeking children and adolescents upon arrival, despite the fact that aspects of physical health tend to push themselves into the foreground [97]. As many young people are expected to show high levels of resilience, it is crucial to not only focus on therapeutic but also preventative actions to reduce psychological distress and promote social function and well-being [98]. These preventative actions range from a collaborative, political efforts to resolve conflict and war, to the training of professionals for work with refugees, and the respect of existing relationships, e.g. between parents and children [91]. Unaccompanied minors should receive special attention as they constitute a high-risk group. Upon arrival in the host country, rapid resolution of asylum claims as well as educational and employment opportunities are seen as important policy measures [6, 91]. Programs should further focus on family cohesion and peer and social support [99]. For therapeutic actions, facilitating an easier access to mental and physical health services may be essential by adapting to the refugees’ help-seeking behaviour, offering practical support and using interpreters [100–102]. Western-based approaches of care should shift to a more ecological culturally sensitive approach, accounting for the complexity of mental distress of refugee and asylum-seeking children and adolescents [94]. Effective core strategies for psychotherapeutic interventions with refugee minors are developing coping strategies, improving self-esteem, and rebuilding identity [103]. Where language barriers are prevalent, art-based interventions should be considered as they have proved to be equally effective as verbal interventions in reducing PTSD symptoms in refugee and asylum-seeking children and adolescents [104].

Our systematic review has several strengths. The greatest strength of our review is the comprehensive search strategy, including hand-searching of reference lists and contacting authors to receive additional data to calculate point prevalence. Moreover, we applied a rigorous methodologically sound systematic review process to address the review question. Nonetheless, we cannot be sure that we have detected every study on prevalence of psychiatric disorders in this population. Upon completion of this review, a new study on Syrian refugee children settled in Turkey was published, with prevalences of 47.9% for depression and 53.2% for anxiety disorders having only a slight impact on the prevalence ranges found for anxiety disorders (3.8–53.2% instead of 3.8–50.5%) [105]. The main weakness of our study is that we did not analyse the results considering gender and age differences or the legal status of children and adolescents (i.e. no differentiation between asylum seekers and refugees). Gender differences in psychiatric disorders were reported to be age-dependant: higher point prevalence was seen in boys than in girls younger than 13 years [90, 106]. Generally, the point prevalence of psychiatric disorders was not reported specifically regarding age and gender as well as the asylum status/immigration label [15] impeding additional analyses.

A considerably large problem is that the evidence base relies mainly on convenience samples not providing information if the investigated population is comparable to a countries young refugee and asylum-seeker population. Further effort needs to be undertaken to conduct representative studies. Currently, we are faced with a dearth of relevant prevalence data for several European countries (e.g. Austria, France, Germany, Hungary, Sweden, Switzerland, and Turkey), leaving the varying legal situations in host countries out of sight. Furthermore, there is an ongoing debate on whether mental illnesses and their symptomatology can be seen as a transcultural phenomenon and if levels of distress are expressed differently in different cultures [11, 107–109]. Some authors called for cautiousness [15]. To investigate potential cultural differences among five different linguistic groups, a large study in the Netherlands used the Harvard Trauma Questionnaire (HTQ) and the Hopkins Symptom Checklist (HSCL-25) for assessing PTSD and anxiety and depression [110]. The results showed good construct validity, indicating that different ways of expressing mental illnesses and their symptomatology might be less a problem than assumed.

Nevertheless, to take planning steps for guaranteeing an effective mental health care, it is important to improve the evidence base on refugees’ mental health in general and in particular for children and adolescents, as they are the most vulnerable group [13, 14, 16]. Research should continue to assess the cultural validity of often applied screening instruments for psychiatric disorders. Further steps should be undertaken to assess the screening instruments sensitivity and specificity to detect psychiatric disorders. To gain more precise estimates of the psychiatric disorder point prevalence, large studies using the same methodology (questionnaires, cut-off-values) based on similar samples regarding age, gender, asylum/immigration status, and countries of origin should be conducted.

In conclusion, the investigated refugee and asylum-seeking children and adolescents were affected by high levels of PTSD, but also by anxiety and depression disorders. As the results were highly heterogeneous, the evidence base could be improved by international methodologically comparable studies with sufficiently large sample sizes drawn randomly among specified refugee populations.

## Electronic supplementary material

Below is the link to the electronic supplementary material.

Supplementary material 1 (DOCX 39 kb)

Supplementary material 2 (DOCX 19 kb)

Supplementary material 3 (DOCX 768 kb)

Supplementary material 4 (DOCX 19 kb)
