# Supplementary material for: Prevalence of mental disorders in young refugees and asylum seekers in European Countries: a systematic review
Source: Eur Child Adolesc Psychiatry. 2018 Aug 27;28(10):1295–310. doi: 10.1007/s00787-018-1215-z (PMC6785579; doi:10.1007/s00787-018-1215-z)
Supplement: Supplementary file 1 — Supplementary material 1 (DOCX 39 kb) [file 787_2018_1215_MOESM1_ESM.docx]

**Additional Material 1:** Search Strategy

Ovid MEDLINE(R) 1946 to October Week 1 2017,

Ovid MEDLINE(R) Epub Ahead of Print October 13, 2017,

Ovid MEDLINE(R) In-Process & Other Non-Indexed Citations October 13, 2017,

Ovid MEDLINE(R) Daily Update October 13, 2017

| **#** | **Search** | **Hints** |
| --- | --- | --- |
| 1 | exp Refugees/ | 8752 |
| 2 | refugee*.ti,ab,kf. | 8763 |
| 3 | asylum.ti,ab,kf. | 2805 |
| 4 | displaced.ti. | 4027 |
| 5 | displaced person*.ab,kf. | 480 |
| 6 | (forced adj (migra* or displace* or immigra*)).ti,ab,kf. | 308 |
| 7 | or/1-6 | 17181 |
| 8 | psychology.fs. | 961891 |
| 9 | exp Mental Health/ | 31270 |
| 10 | exp Mental Disorders/ | 1169106 |
| 11 | exp Stress, Psychological/ | 117967 |
| 12 | exp Depression/ | 103790 |
| 13 | exp Anxiety/ | 75179 |
| 14 | exp Substance-Related Disorders/ | 266752 |
| 15 | mental health.ti,ab,kf. | 124998 |
| 16 | psycholog*.ti,ab,kf. | 253312 |
| 17 | stress.ti,ab,kf. | 644045 |
| 18 | distress*.ti,ab,kf. | 108944 |
| 19 | ptsd.ti,ab,kf. | 20165 |
| 20 | anxiety.ti,ab,kf. | 163547 |
| 21 | depress*.ti,ab,kf. | 421225 |
| 22 | phobia*.ti,ab,kf. | 8788 |
| 23 | panic.ti,ab,kf. | 14213 |
| 24 | bipolar.ti,ab,kf. | 58053 |
| 25 | schizophren*.ti,ab,kf. | 119930 |
| 26 | drug?.ti,ab,kf. | 1512870 |
| 27 | (substance? adj1 (abuse or illegal or illicit)).ti,ab,kf. | 25424 |
| 28 | alcohol*.ti,ab,kf. | 308275 |
| 29 | tobacco.ti,ab,kf. | 91660 |
| 30 | smoking.ti,ab,kf. | 204824 |
| 31 | or/8-30 | 4443602 |
| 32 | 7 and 31 | 5935 |
| 33 | adolescent/ or exp child/ | 2913316 |
| 34 | Minors/ | 2539 |
| 35 | child*.ti,ab,kf. | 1324632 |
| 36 | minor?.ti,ab,kf. | 215413 |
| 37 | youth?.ti,ab,kf. | 65911 |
| 38 | adolescen*.ti,ab,kf. | 261553 |
| 39 | young.ti,ab,kf. | 435057 |
| 40 | underage*.ti,ab,kf. | 1054 |
| 41 | or/33-40 | 3752812 |
| 42 | 32 and 41 | 2251 |
| 43 | exp Europe/ | 1346283 |
| 44 | europ*.af. | 1086203 |
| 45 | (Albania* or Andorra* or Armenia* or Austria* or Azerbaijan*).af. | 263750 |
| 46 | (Belarus* or Belgi* or Bosnia* or Herzegov* or Bulgaria*).af. | 365444 |
| 47 | (Croatia* or Cypr* or Czech).af. | 234151 |
| 48 | (Denmark or danish).af. | 380235 |
| 49 | Estonia*.af. | 9868 |
| 50 | (Finland or finnish or France or french).af. | 1754121 |
| 51 | (Georgia* or German* or Greece or greek).af. | 3348428 |
| 52 | Hungar*.af. | 121002 |
| 53 | (Iceland* or Ireland or irish or Italy or italian).af. | 1561527 |
| 54 | (Kazak* or Kosov*).af. | 10039 |
| 55 | (Latvia* or Liechtenstein* or Lithuania* or Luxembourg*).af. | 21190 |
| 56 | (Macedonia* or Malta or maltese or Moldov* or Monac* or Montenegr*).af. | 26679 |
| 57 | (Netherlands or dutch or Norway or norwegian).af. | 2200229 |
| 58 | (Poland or polish or Portug*).af. | 575347 |
| 59 | (Romania* or Russia*).af. | 872212 |
| 60 | (San Marino or Serbia* or Slovakia* or Slovenia* or Spain or spanish or Sweden or swedish or Switzerland or swiss).af. | 2240757 |
| 61 | (Turkey or turkish).af. | 240156 |
| 62 | (Ukrain* or United Kingdom or britain or british or Vatican).af. | 1504469 |
| 63 | Balkan*.af. | 6629 |
| 64 | Mediterranean.af. | 43862 |
| 65 | Scandinavian.af. | 86570 |
| 66 | (Transcaucas* or Trans-caucas*).af. | 220 |
| 67 | or/43-66 | 13710306 |
| 68 | 42 and 67 | 1244 |
| 69 | limit 68 to yr="1990 -Current" | 1186 |
| 70 | remove duplicates from 69 | 1077 |

**PsycINFO (Ebsco):** 16 October 2017

| **#** | **Query** | **Limiters/Expanders** | **Hints** |
| --- | --- | --- | --- |
| S1 | DE "Refugees" OR DE "Asylum Seeking" | Search modes - Find all my search terms | 5,390 |
| S2 | refugee* | Search modes - Find all my search terms | 7,566 |
| S3 | asylum | Search modes - Find all my search terms | 3,380 |
| S4 | TI displaced | Search modes - Find all my search terms | 574 |
| S5 | "displaced person*" | Search modes - Find all my search terms | 359 |
| S6 | forced W0 (migra* OR displace* OR immigra*) | Search modes - Find all my search terms | 450 |
| S7 | S1 OR S2 OR S3 OR S4 OR S5 OR S6 | Search modes - Find all my search terms | 10,644 |
| S8 | DE "Mental Health" | Search modes - Find all my search terms | 60,132 |
| S9 | DE "Mental Disorders" | Search modes - Find all my search terms | 120,71 |
| S10 | DE "Stress" OR DE "Post-Traumatic Stress" OR DE "Psychological Stress" | Search modes - Find all my search terms | 61,192 |
| S11 | DE "Major Depression" OR DE "Depression (Emotion)" | Search modes - Find all my search terms | 130,286 |
| S12 | DE "Generalized Anxiety Disorder" OR DE "Panic Disorder" OR DE "Anxiety" OR DE "Posttraumatic Stress Disorder" OR DE "Complex PTSD" OR DE "DESNOS" OR DE "Anxiety Disorders" OR DE "Acute Stress Disorder" OR DE "Phobias" | Search modes - Find all my search terms | 127,446 |
| S13 | DE "Emotional Trauma" | Search modes - Find all my search terms | 14,813 |
| S14 | DE "Psychiatric Symptoms" OR DE "Bipolar Disorder" | Search modes - Find all my search terms | 44,815 |
| S15 | DE "Psychopathology" OR DE "Adolescent Psychopathology" OR DE "Child Psychopathology" | Search modes - Find all my search terms | 34,391 |
| S16 | DE "Schizophrenia" OR DE "Acute Schizophrenia" OR DE "Catatonic Schizophrenia" OR DE "Childhood Schizophrenia" OR DE "Paranoid Schizophrenia" OR DE "Process Schizophrenia" OR DE "Schizophrenia (Disorganized Type)" OR DE "Schizophreniform Disorder" OR DE "Undifferentiated Schizophrenia" | Search modes - Find all my search terms | 92,997 |
| S17 | DE "Distress" | Search modes - Find all my search terms | 18,927 |
| S18 | DE "Adjustment Disorders" OR DE "Behavior Problems" | Search modes - Find all my search terms | 29,655 |
| S19 | DE "Addiction" OR DE "Alcoholism" OR DE "Drug Addiction" | Search modes - Find all my search terms | 59,095 |
| S20 | DE "Behavior Disorders" OR DE "Drug Usage" OR DE "Alcohol Abuse" OR DE "Drug Dependency" | Search modes - Find all my search terms | 52,689 |
| S21 | DE "Tobacco Smoking" | Search modes - Find all my search terms | 28,095 |
| S22 | DE "Substance Use Disorder" | Search modes - Find all my search terms | 5,162 |
| S23 | "mental health" | Search modes - Find all my search terms | 507,073 |
| S24 | psycholog* | Search modes - Find all my search terms | 2,219,903 |
| S25 | stress | Search modes - Find all my search terms | 244,415 |
| S26 | distress* | Search modes - Find all my search terms | 65,904 |
| S27 | ptsd | Search modes - Find all my search terms | 30,383 |
| S28 | anxiety | Search modes - Find all my search terms | 233,124 |
| S29 | depress* | Search modes - Find all my search terms | 324,075 |
| S30 | phobia* | Search modes - Find all my search terms | 15,844 |
| S31 | panic | Search modes - Find all my search terms | 16,544 |
| S32 | bipolar | Search modes - Find all my search terms | 43,762 |
| S33 | schizophren* | Search modes - Find all my search terms | 137,495 |
| S34 | drug OR drugs | Search modes - Find all my search terms | 409,126 |
| S35 | substance* N0 (abuse OR illegal OR illicit) | Search modes - Find all my search terms | 95,208 |
| S36 | alcohol* | Search modes - Find all my search terms | 168,376 |
| S37 | tobacco | Search modes - Find all my search terms | 38,076 |
| S38 | smoking | Search modes - Find all my search terms | 51,629 |
| S39 | S8 OR S9 OR S10 OR S11 OR S12 OR S13 OR S14 OR S15 OR S16 OR S17 OR S18 OR S19 OR S20 OR S21 OR S22 OR S23 OR S24 OR S25 OR S26 OR S27 OR S28 OR S29 OR S30 OR S31 OR S32 OR S33 OR S34 OR S35 OR S36 OR S37 OR S38 | Search modes - Find all my search terms | 2,959,278 |
| S40 | S7 AND S39 | Search modes - Find all my search terms | 8,018 |
| S41 | (ZG "adolescence (13-17 yrs)") or (ZG "childhood (birth-12 yrs)") or (ZG "preschool age (2-5 yrs)") or (ZG "school age (6-12 yrs)") | Search modes - Find all my search terms | 714,175 |
| S42 | child* OR minor OR minors OR youth* OR adolescen* OR young OR underage | Search modes - Find all my search terms | 1,180,457 |
| S43 | S41 OR S42 | Search modes - Find all my search terms | 1,323,212 |
| S44 | S40 AND S43 | Search modes - Find all my search terms | 2,936 |
| S45 | TX Albania* OR Andorra* OR Armenia* OR Austria* OR Azerbaijan* OR Belarus* OR Belgi* OR Bosnia* OR Herzegov* OR Bulgaria* OR Croatia* OR Cypr* OR Czech OR Denmark OR danish OR Estonia* OR Finland OR finnish OR France OR french OR Georgia* OR German* OR Greece OR greek OR Hungar* OR Iceland* OR Ireland OR irish OR Italy OR italian OR Kazak* OR Kosov* OR Latvia* OR Liechtenstein* OR Lithuania* OR Luxembourg* OR Macedonia* OR Malta OR maltese OR Moldov* OR Monac* OR Montenegr* OR Netherlands OR dutch OR Norway OR norwegian OR Poland OR polish OR Portug* OR Romania* OR Russia* OR San Marino OR Serbia* OR Slovakia* OR Slovenia* OR Spain OR spanish OR Sweden OR swedish OR Switzerland OR swiss OR Turkey OR turkish OR Ukrain* OR United Kingdom OR britain OR british OR Vatican | Search modes - Boolean/Phrase | 1,221,577 |
| S46 | TX europ* OR balkan* OR Mediterranean OR Scandinavian OR Transcaucas* OR Trans-caucas* | Search modes - Find all my search terms | 144,725 |
| S47 | S45 OR S46 | Search modes - Find all my search terms | 1,268,128 |
| S48 | S44 AND S47 | Search modes - Find all my search terms | 1,293 |
| S49 | S48 | Limiters - Published Date: 19900101-20171231 | 1,198 |

**CINAHL (Ebsco):** 16 October 2017

| **#** | **Query** | **Limiters/Expanders** | **Hints** |
| --- | --- | --- | --- |
| S1 | (MH "Refugees") | Search modes - Find all my search terms | 3,996 |
| S2 | refugee* | Search modes - Find all my search terms | 4,697 |
| S3 | asylum | Search modes - Find all my search terms | 1,109 |
| S4 | TI displaced | Search modes - Find all my search terms | 751 |
| S5 | "displaced person*" | Search modes - Find all my search terms | 115 |
| S6 | forced W0 (migra* OR displace* OR immigra*) | Search modes - Find all my search terms | 88 |
| S7 | S1 OR S2 OR S3 OR S4 OR S5 OR S6 | Search modes - Find all my search terms | 5,845 |
| S8 | MH "Mental Health" | Search modes - Find all my search terms | 15,422 |
| S9 | MH "Mental Disorders+" | Search modes - Find all my search terms | 296,625 |
| S10 | (MH "Stress") OR (MH "Stress, Psychological") OR (MH "Minority Stress") | Search modes - Find all my search terms | 29,655 |
| S11 | (MH "Anxiety") | Search modes - Find all my search terms | 19,751 |
| S12 | (MH "Substance Dependence+") | Search modes - Find all my search terms | 51,316 |
| S13 | psycholog* | Search modes - Find all my search terms | 150,531 |
| S14 | stress | Search modes - Find all my search terms | 101,018 |
| S15 | distress* | Search modes - Find all my search terms | 30,559 |
| S16 | ptsd | Search modes - Find all my search terms | 4,362 |
| S17 | anxiety | Search modes - Find all my search terms | 44,383 |
| S18 | depress* | Search modes - Find all my search terms | 87,929 |
| S19 | phobia* | Search modes - Find all my search terms | 1,046 |
| S20 | panic | Search modes - Find all my search terms | 2,222 |
| S21 | bipolar | Search modes - Find all my search terms | 7,482 |
| S22 | schizophren* | Search modes - Find all my search terms | 13,419 |
| S23 | drug OR drugs | Search modes - Find all my search terms | 442,099 |
| S24 | substance* N0 (abuse OR illegal OR illicit) | Search modes - Find all my search terms | 31,52 |
| S25 | alcohol* | Search modes - Find all my search terms | 52,166 |
| S26 | tobacco OR smoking | Search modes - Find all my search terms | 61,618 |
| S27 | S8 OR S9 OR S10 OR S11 OR S12 OR S13 OR S14 OR S15 OR S16 OR S17 OR S18 OR S19 OR S20 OR S21 OR S22 OR S23 OR S24 OR S25 OR S26 | Search modes - Find all my search terms | 898,514 |
| S28 | (MH "Child+") OR (MH "Adolescence+") | Search modes - Find all my search terms | 493,277 |
| S29 | child* OR minor OR minors OR youth* OR adolescen* OR young OR underage | Search modes - Find all my search terms | 612,634 |
| S30 | S28 OR S29 | Search modes - Find all my search terms | 647,51 |
| S31 | TX Albania* OR Andorra* OR Armenia* OR Austria* OR Azerbaijan* OR Belarus* OR Belgi* OR Bosnia* OR Herzegov* OR Bulgaria* OR Croatia* OR Cypr* OR Czech OR Denmark OR danish OR Estonia* OR Finland OR finnish OR France OR french OR Georgia* OR German* OR Greece OR greek OR Hungar* OR Iceland* OR Ireland OR irish OR Italy OR italian OR Kazak* OR Kosov* OR Latvia* OR Liechtenstein* OR Lithuania* OR Luxembourg* OR Macedonia* OR Malta OR maltese OR Moldov* OR Monac* OR Montenegr* OR Netherlands OR dutch OR Norway OR norwegian OR Poland OR polish OR Portug* OR Romania* OR Russia* OR San Marino OR Serbia* OR Slovakia* OR Slovenia* OR Spain OR spanish OR Sweden OR swedish OR Switzerland OR swiss OR Turkey OR turkish OR Ukrain* OR United Kingdom OR britain OR british OR Vatican | Search modes - Boolean/Phrase | 384,75 |
| S32 | TX europ* OR balkan* OR Mediterranean OR Scandinavian OR Transcaucas* OR Trans-caucas* | Search modes - Find all my search terms | 1,078,988 |
| S33 | S31 OR S32 | Search modes - Find all my search terms | 1,174,941 |
| S34 | S7 AND S27 AND S30 AND S33 | Search modes - Find all my search terms | 344 |
| S35 | S34 | Limiters - Published Date: 19900101-20171231 | 344 |

**PubMED (non-Medline content)**: 16 October 2017

| **Search** | **Query** | **Hints** |
| --- | --- | --- |
| #1 | Search refugee*[tw] | 11055 |
| #2 | Search asylum[tw] | 2623 |
| #3 | Search displaced[ti] | 3755 |
| #4 | Search displaced person*[tw] | 517 |
| #5 | Search forced migra*[tw] OR forced immigra*[tw] OR forced displac*[tw] | 293 |
| #6 | Search #5 OR #4 OR #3 OR #2 OR #1 | 16089 |
| #7 | Search mental health[tw] | 151441 |
| #8 | Search psycholog*[tw] | 1263251 |
| #9 | Search stress[tw] | 749842 |
| #10 | Search distress*[tw] | 116410 |
| #11 | Search ptsd[tw] | 18827 |
| #12 | Search anxiety[tw] | 193528 |
| #13 | Search depress*[tw] | 469366 |
| #14 | Search phobia*[tw] | 8492 |
| #15 | Search panic[tw] | 14812 |
| #16 | Search bipolar[tw] | 69504 |
| #17 | Search schizophren*[tw] | 132807 |
| #18 | Search addict*[tw] | 57064 |
| #19 | Search drug[tw] OR drugs[tw] | 5262237 |
| #20 | Search illegal substance*[tw] OR substance abuse[tw] OR illicit substance*[tw] | 47825 |
| #21 | Search alcohol*[tw] | 368521 |
| #22 | Search tobacco[tw] | 108971 |
| #23 | Search smoking[tw] | 245394 |
| #24 | Search #23 OR #22 OR #21 OR #20 OR #19 OR #18 OR #17 OR #16 OR #15 OR #14 OR #13 OR #12 OR #11 OR #10 OR #9 OR #8 OR #7 | 7557137 |
| #25 | Search (#6 AND #24) | 5743 |
| #26 | Search child*[tw] OR minor[tw] OR minors[tw] OR youth*[tw] OR adolescen*[tw] OR young[tw] OR underage*[tw] | 3784110 |
| #27 | Search (#25 AND #26) | 2372 |
| #28 | Search pubmednotmedline [sb] OR pmcbook OR (publisher [sb] AND (pubstatusnihms OR pubstatuspmcsd)) | 2107427 |
| #29 | Search (#27 AND #28) | 75 |
| #30 | Search (#29) AND ("1990"[Date - Publication] : "3000"[Date - Publication]) | 73 |

**Scopus:** 17 October 2017

| **Search** | **Query** | **Hints** |
| --- | --- | --- |
| #1 | Search refugee*[tw] | 11055 |
| #2 | Search asylum[tw] | 2623 |
| #3 | Search displaced[ti] | 3755 |
| #4 | Search displaced person*[tw] | 517 |
| #5 | Search forced migra*[tw] OR forced immigra*[tw] OR forced displac*[tw] | 293 |
| #6 | Search #5 OR #4 OR #3 OR #2 OR #1 | 16089 |
| #7 | Search mental health[tw] | 151441 |
| #8 | Search psycholog*[tw] | 1263251 |
| #9 | Search stress[tw] | 749842 |
| #10 | Search distress*[tw] | 116410 |
| #11 | Search ptsd[tw] | 18827 |
| #12 | Search anxiety[tw] | 193528 |
| #13 | Search depress*[tw] | 469366 |
| #14 | Search phobia*[tw] | 8492 |
| #15 | Search panic[tw] | 14812 |
| #16 | Search bipolar[tw] | 69504 |
| #17 | Search schizophren*[tw] | 132807 |
| #18 | Search addict*[tw] | 57064 |
| #19 | Search drug[tw] OR drugs[tw] | 5262237 |
| #20 | Search illegal substance*[tw] OR substance abuse[tw] OR illicit substance*[tw] | 47825 |
| #21 | Search alcohol*[tw] | 368521 |
| #22 | Search tobacco[tw] | 108971 |
| #23 | Search smoking[tw] | 245394 |
| #24 | Search #23 OR #22 OR #21 OR #20 OR #19 OR #18 OR #17 OR #16 OR #15 OR #14 OR #13 OR #12 OR #11 OR #10 OR #9 OR #8 OR #7 | 7557137 |
| #25 | Search (#6 AND #24) | 5743 |
| #26 | Search child*[tw] OR minor[tw] OR minors[tw] OR youth*[tw] OR adolescen*[tw] OR young[tw] OR underage*[tw] | 3784110 |
| #27 | Search (#25 AND #26) | 2372 |
| #28 | Search pubmednotmedline [sb] OR pmcbook OR (publisher [sb] AND (pubstatusnihms OR pubstatuspmcsd)) | 2107427 |
| #29 | Search (#27 AND #28) | 75 |
| #30 | Search (#29) AND ("1990"[Date - Publication] : "3000"[Date - Publication]) | 73 |
