# Supplementary material for: Prevalence of mental disorders in young refugees and asylum seekers in European Countries: a systematic review
Source: Eur Child Adolesc Psychiatry. 2018 Aug 27;28(10):1295–310. doi: 10.1007/s00787-018-1215-z (PMC6785579; doi:10.1007/s00787-018-1215-z)
Supplement: Supplementary file 2 — Supplementary material 2 (DOCX 19 kb) [file 787_2018_1215_MOESM2_ESM.docx]

**Additional Material 2:** Mean and standard deviations for studies not reporting prevalences for relevant outcomes

| **Study** | **Outcome** | **Results, M (SD)** | | | **Sample Size** | **Questionnaires** |
| --- | --- | --- | --- | --- | --- | --- |
| Barghadouch et al. (2016) | AD*, ND*, PD* | Adjusted RR (reference = danish-born adolescents)  AD: 0.73 (95% CI: 0.55-0.89)  ND: 1.21 (95% CI: 1.08-1.37)  PD: 1.72 (95% CI: 1.33-2.22) | | | 15,264 | Register-based study |
| Bronstein et al. (2012) | A, D, EBP, EP*, IP, PTSD | EP: 14.81 (4.2) | | | 222 | EP: HSCL-37A |
| Gavranidou et al. (2008) | A*, EBP*, | **Boys:**  A: 6.6 (4.6) EBP: 39.3 (19.0) | | **Girls:**  A: 6.8 (4.6) EBP: 39.4 (18.0) | Boys: 32  Girls: 23 | A: YSR  EBP: YSR |
| Huemer et al. (2011) | A*, D, PTSD, S | A: 3 (0.59) | | | 41 | A: WAI |
| Kocijan-Hercigonja et al. (1998) | A*, D*, PSYCom* | A: 5.58 (2.01)  D: 5.08 (2.94)  PSYCom: 6.05 (2.48) | | | 35 | Anxiety Scale  Depression Scale  Questionnaire of Psychosomatic Symptoms |
| Oppedal et al. (2015) | D*, PTSD | D: 20.89 (9.49) | | | 859 | D: CES-D |
| Reijneveld et al. (2005) | A*, D*, EBP*, PTSD* | **Restricted Campus**  A: 22.9 (6.1)  D: 36.4 (8.2)  EBP: 59.3 (13.1)  PTSD: 56.1 (10.4) | **Routine Campus**  A: 19.4 (4.2)  D: 34.0 (7.2)  EBP: 53.4 (10.5)  PTSD: 51.5 (11.6) | | Restricted Campus: 69  Routine Campus: 46-51 | A: HSCL-25  D: HSCL-25  EBP: HSCL-25  PTSD: RATS |
| Sabuncuoglu et al. 2002 | D* | D: 11.57 (5.96) | | | 19 | D: CDI |
| Sikic et al. (1997) | A*, D* | Standardized z-values: A: 0.74 (1.13)  D: 0.2 (1.24) | | | 669 | A: CPRS  D: CPRS |
| Yurtbay et al. (2003) | A, D* | D:  children: 15.7 (NR)  adolescents: 17.6 (NR) | | | children: 150 adolescents: 100 | D:  Children: CDI  Adolescents: Beck Depression Inventory |

**Abbreviations:** A, Anxiety; AD, affective disorder; CES-D, Center for Epidemiological Studies Depression Scale; CDI, Children’s Depression Inventory; CPRS, Children’s Psychiatric Rating Scale; D, Depression; EBP, Emotional and behavioural problems; EP, Externalizing problems; HSCL-25, Hopkins Symptom Checklist 25; HSCL-37A, Hopkins Symptom Checklist 37A; M, Mean; n, sample size; ND, neurotic disorder; NR, not reported; PD, psychotic disorder; PSYCom, psychiatric complaints; PTSD, posttraumatic stress disorder; RATS, Reactions of Adolescents to Traumatic Stress Questionnaire; S, suicidal ideation and behaviour; SD, standard deviation; WAI, Weinberger Adjustment Inventory; YSR, Youth Self Report Questionnaire;

**Notes:**
* mean and standard deviation but no point prevalences are reported
