# Supplementary material for: Prevalence of mental disorders in young refugees and asylum seekers in European Countries: a systematic review
Source: Eur Child Adolesc Psychiatry. 2018 Aug 27;28(10):1295–310. doi: 10.1007/s00787-018-1215-z (PMC6785579; doi:10.1007/s00787-018-1215-z)
Supplement: Supplementary file 3 — Supplementary material 3 (DOCX 768 kb) [file 787_2018_1215_MOESM3_ESM.docx]

**Additional material 3:** Results of the Meta-Analyses

**
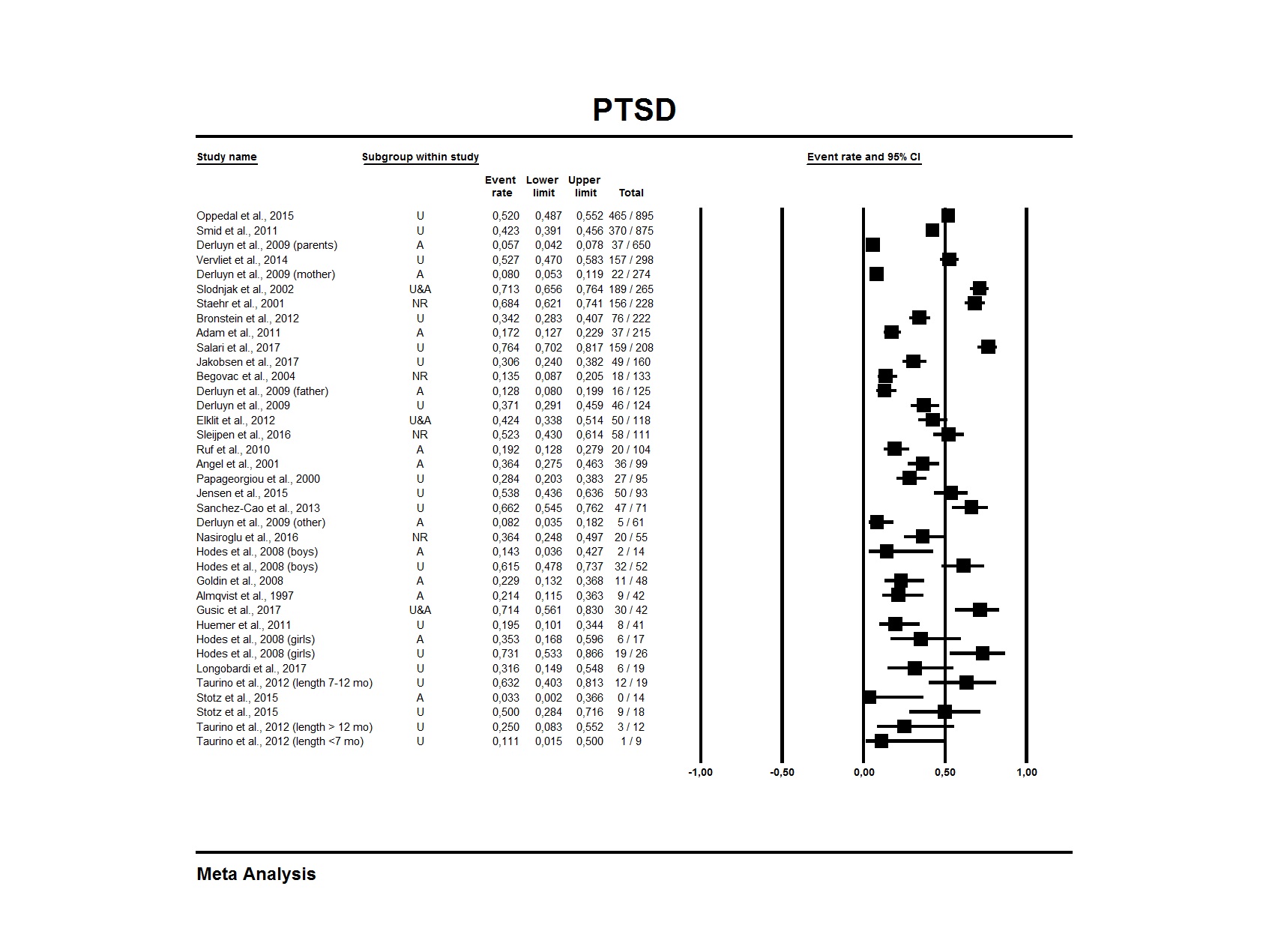
**

Figure 1 Meta-Analysis of the reported point prevalences of PTSD. Random effects meta-analysis; I^2^=96%, Tau^2^=0.88

Abbreviations: A, accompanied minors; NR, not reported; U, unaccompanied minors

**
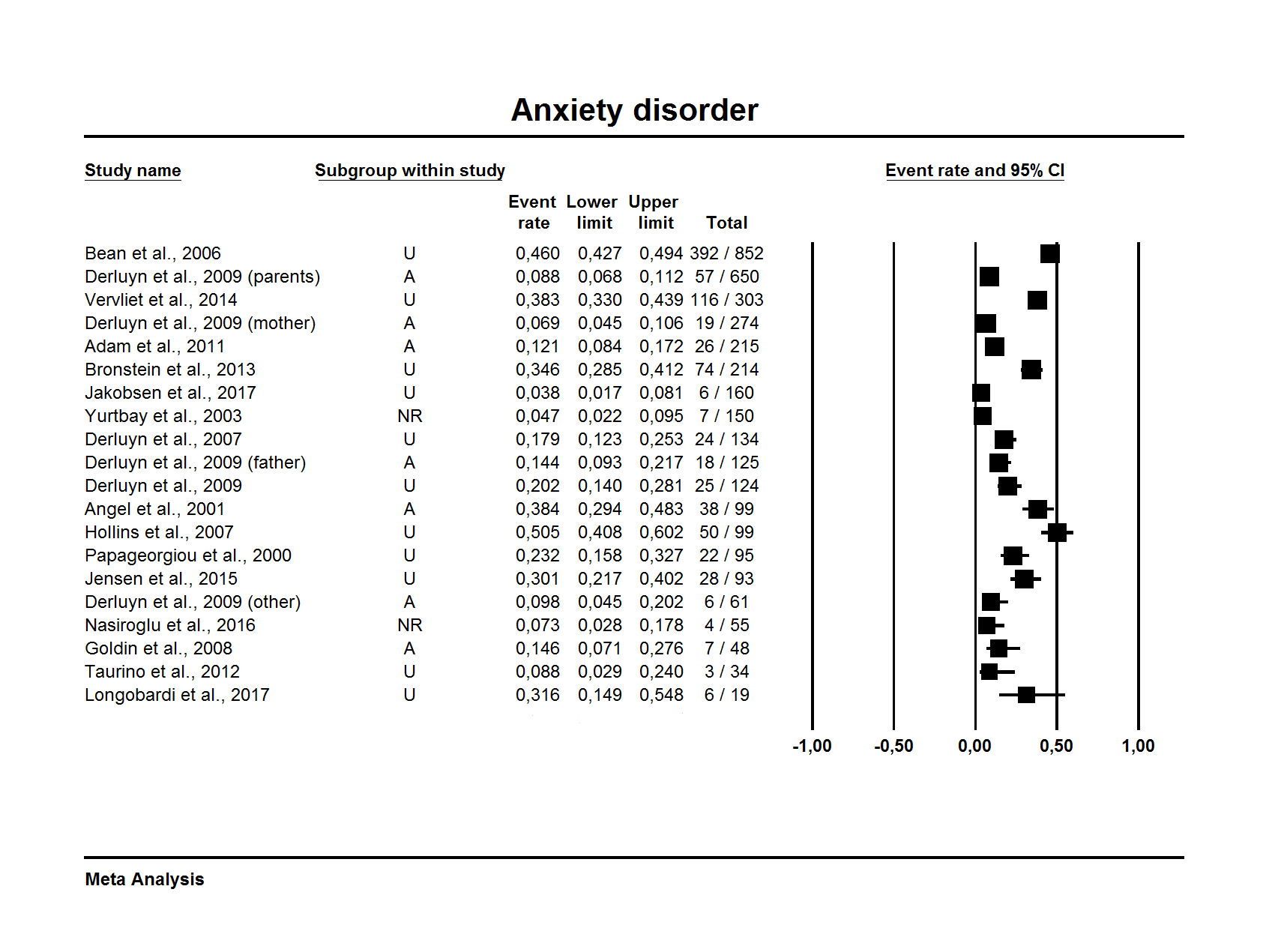
**

Figure 2 Meta-Analysis of the reported point prevalences of anxiety disorders. Random effects meta-analysis; I^2^=96%, Tau^2^=0.88

Abbreviations: A, accompanied minors; NR, not reported; U, unaccompanied minors


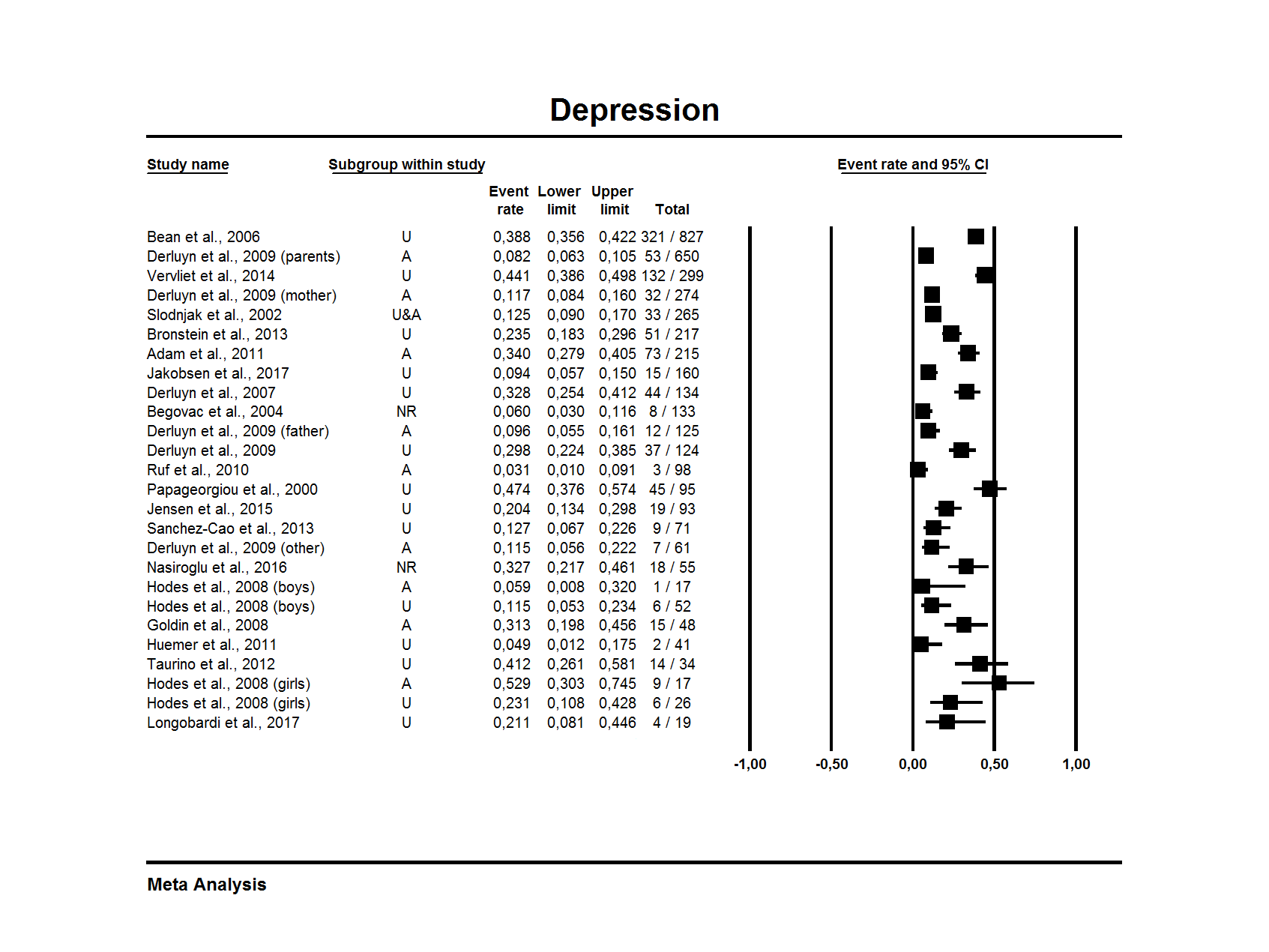


Figure 3 Meta-Analysis of the reported point prevalences of depression. Random effects meta-analysis; I^2^=94%, Tau^2^=0.96

Abbreviations: A, accompanied minors; NR, not reported; U, unaccompanied minors


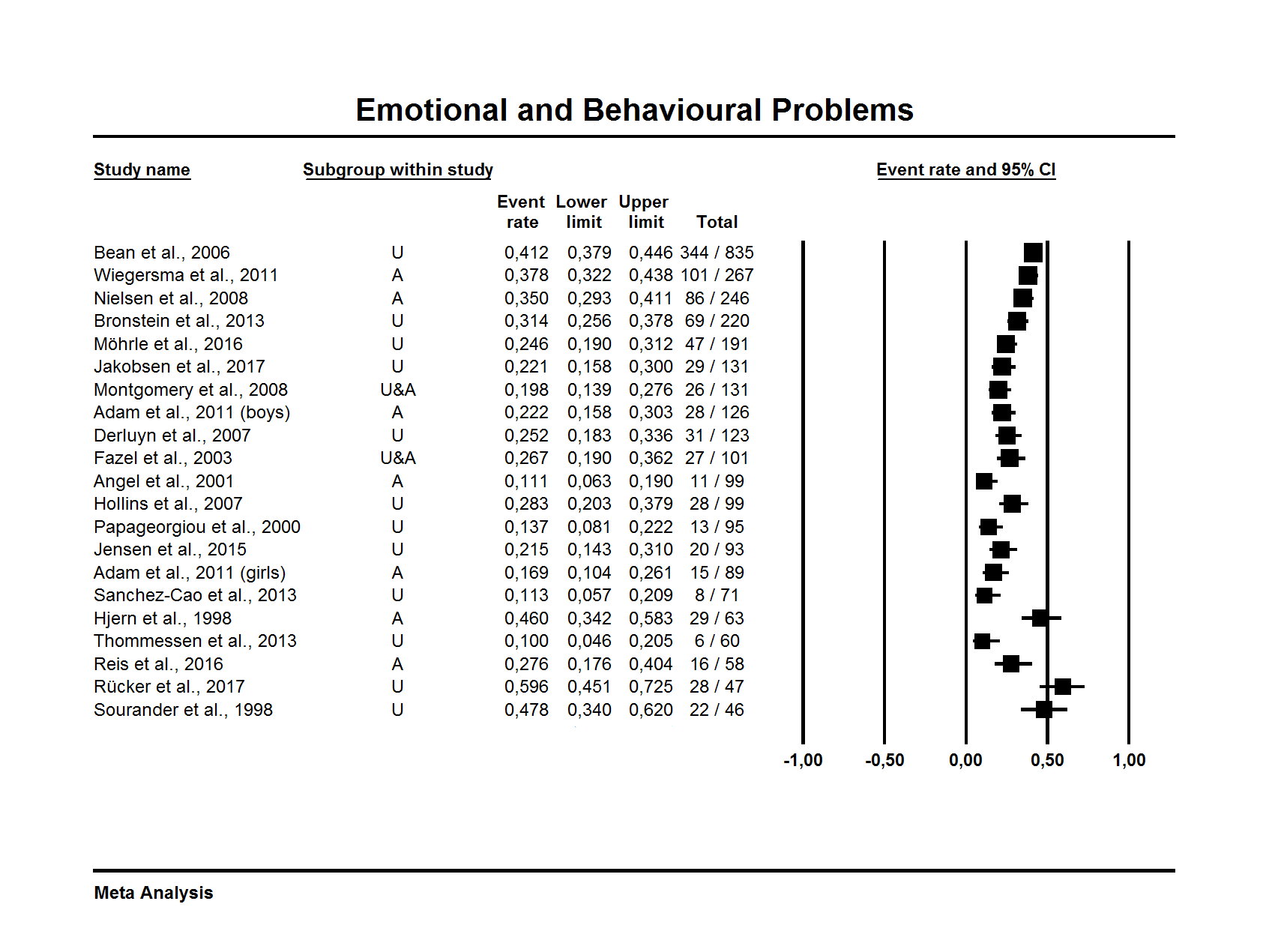


Figure 4 Meta-Analysis of the reported point prevalences of emotional and behavioural problems. Random effects meta-analysis; I^2^=87%, Tau^2^=0.25

Abbreviations: A, accompanied minors; U, unaccompanied minors


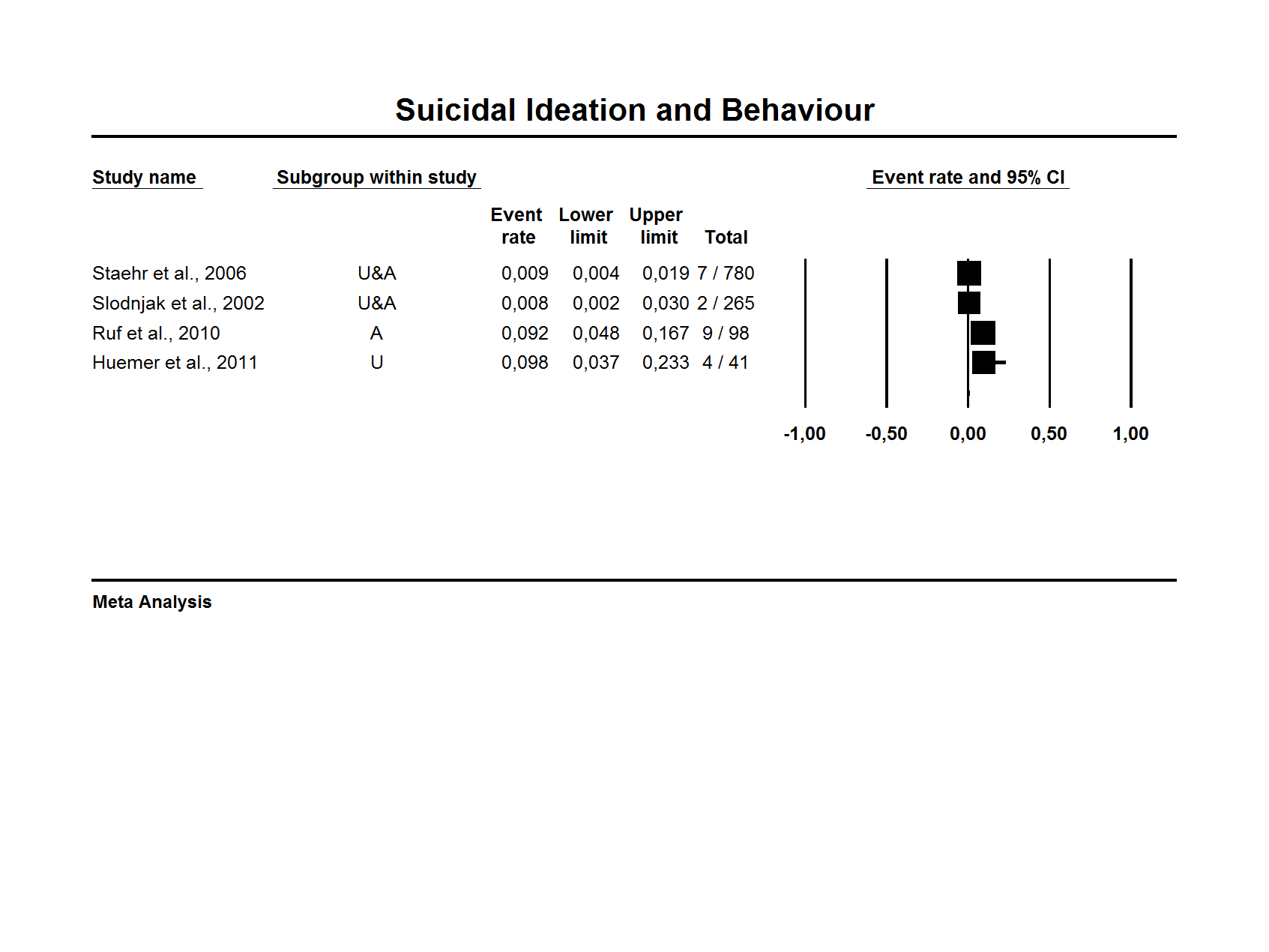


Figure 5 Meta-Analysis of the reported point prevalences of suicidal ideation and behaviour. Random effects meta-analysis; I^2^=90%, Tau^2^=1.96

Abbreviations: A, accompanied minors, U, unaccompanied minors
