# Supplementary material for: Prevalence of mental disorders in young refugees and asylum seekers in European Countries: a systematic review
Source: Eur Child Adolesc Psychiatry. 2018 Aug 27;28(10):1295–310. doi: 10.1007/s00787-018-1215-z (PMC6785579; doi:10.1007/s00787-018-1215-z)
Supplement: Supplementary file 4 — Supplementary material 4 (DOCX 19 kb) [file 787_2018_1215_MOESM4_ESM.docx]

**Additional Material 4:** List of instruments

| **psychiatric disorder** | **abbreviation** | **name of tool** |
| --- | --- | --- |
| Affective Disorder | K-SADS-PL | Kiddie Schedule for Affective Disorders and Schizophrenia- Present and Lifetime |
| Anxiety | HAM-A | Hamilton Anxiety Scale |
| Anxiety | RCMAS | Revised Children's Manifest Anxiety Scale |
| Anxiety | STAI | State-Trait Anxiety Inventory |
| Anxiety, Depression | CPRS | Children`s Psychiatric Rating Scale |
| Anxiety, Depression and Somatization | SCWP | Scales for Children Afflicted by War and Persecution |
| Anxiety, Depression, PTSD | TSCC | Trauma Symptom Checklist for Children |
| Depression | BDSR | Birleson Depression Self-Rating Scale |
| Depression | CDI | Children's Depression Inventory |
| Depression | CES-D | Center for Epidemiological Studies Depression Scale |
| Depression | DSRSC | Depression Self-Rating Scale for Children |
| Depression | HAM-D | Hamilton Rating Scale for Depression |
| Emotional and Behavioural Difficulties (Internalizing and Externalizing Problems) | CBCL 4-18 | Child Behavior Checklist |
| Emotional and Behavioural Difficulties (Internalizing and Externalizing Problems) | SDQ | Strenght and Difficulties Questionnaire |
| Emotional and Behavioural Difficulties (Internalizing and Externalizing Problems) | YABC | Young Adult Behavior Checklist |
| Emotional and Behavioural Difficulties (Internalizing and Externalizing Problems) | YASR | Young Adult Self Report |
| Emotional and Behavioural Difficulties (Internalizing and Externalizing Problems) | YSR 11-18 | Youth Self Report |
| Emotional Distress | HSCL-25 | Hopkins Symptom Checklist-25 |
| Emotional Distress | RHS-15 | Refugee Health Screener 15 |
| Emotional Distress, Internalizing and Externalizing Problem Behaviors | HSCL-37A | Hopkins Symptom Checklist-37A |
| Psychiatric Disorder | Cederblad Child Symptom Scale | Cederblad's Child Symptom Scale |
| Psychiatric Disorder | M.I.N.I. Kid | Mini-International Neuropsychiatric Interview for children and adolescents |
| Psychological Ill Health | GHQ-8 | General Health Questionnaire |
| PTSD | CPSS | Child Posttraumatic Stress Disorder Symptom Scale |
| PTSD | CRIES-13 | Children's Revised Impact of Event Scale |
| PTSD | CRIES-8 | Children`s Revised Impact of Event Scale |
| PTSD | DAS | Scheda di rilevazione die sintomi riferiti al Disturbo Acuto da Stress o Disturbo Post Traumatico da Stress (PTSD) |
| PTSD | HTQ | Harvard Trauma Questionnaire |
| PTSD | IES | Impact of Event Scale |
| PTSD | IWRITE | Impact of War-related Trauma Events |
| PTSD | PTSRC | Macksound`s Posttraumatic Stress Reaction Checklist |
| PTSD | PTSR-C Questionnaire | Questionnaire for the Examination of Posttraumatic Stress Reactions in Children |
| PTSD | PTSS16 | Post Traumatic Symptom Scale-16 |
| PTSD | RATS | Reactions of Adolescents to Traumatic Stress Questionnaire |
| PTSD | UCLA CPTS-RI | University of California at Los Angeles PTSD Reaction Index for the Diagnostic and Statistical Manual of Mental Disorders |
| Social Emotional Adjustment (Repressive Defensiveness, Denial of Distress, Restraint and Distress) | WAI | Weinberger Adjustment Inventory |
